# Effect of Sb and Zn Addition on the Microstructures and Tensile Properties of Sn–Bi-Based Alloys

**DOI:** 10.3390/ma15030884

**Published:** 2022-01-24

**Authors:** Akira Yamauchi, Masashi Kurose

**Affiliations:** National Institute of Technology, Gunma College, 580 Toriba-machi, Maebashi 371-8530, Japan; kurose@gunma-ct.ac.jp

**Keywords:** Sn–Bi-based alloy, superplasticity, tensile property, deformation, low-melting-point solder

## Abstract

The tensile behavior of Sn–Bi–Cu and Sn–Bi–Ni alloys has been widely investigated. Reportedly, the addition of small amounts of a third element can refine the microstructures of the eutectic Sn-58mass% Bi solder and improve its ductility. However, the superplasticity mechanism of Sn-based alloys has not been clearly established. Therefore, in this study, the effects of Sb and Zn addition on the microstructures and tensile properties of Sn–Bi-based alloys were investigated. The alloys were subjected to tensile tests under various strain rates and temperatures. We found that Zn- and Sb-added Sn–Bi-based alloys demonstrated superplastic deformation at high temperatures and low strain rates. Sb addition significantly affected the elongation of the Sn–Bi–Sb alloys because the metal dissolves in both the primary Sn phase and the eutectic Sn–Bi matrix. The segregation of Zn and formation of needle-like Zn particles at the eutectic Sn–Bi phase boundary affected the superplastic deformation of the alloys. The deformation of the Sn–40Bi-based alloys at high temperatures and low strain rates led to dynamic recovery, dynamic recrystallization, and/or grain boundary slip because of the accumulation of voids.

## 1. Introduction

The use of Pb and Pb-containing products has been banned in many countries because of their harmful effects on the human body and environment [1,2]. Therefore, several types of Pb-free solder alloys, including Sn-58mass%Bi (412 K) [3], Sn-9mass%Zn (471 K) [4], Sn-0.7mass%Cu (500 K) [5], Sn-3.5mass%Ag (494 K) [6], Sn-70mass%Au (553 K) [7], Sn-35mass%Bi-1mass%Ag (460 K) [8], Sn-8mass%Zn-3mass%Bi (461 K) [9], and Sn-3mass%–0.5mass%Cu (490 K) [10] systems, have been developed to replace Sn-37mass%Pb (456 K) systems in the electronic packaging industries. For example, Sn-3.0mass% Ag-0.5mass% Cu (SAC305), a Pb-free solder, was developed as a substitute for Sn–Pb solder because of its high strength and joint reliability. However, SAC305 is unsuitable for some low-heat-resistance components because its melting temperature, at approximately 494 K, is higher than that of the Sn–Pb eutectic solder (456 K).

Eutectic Sn-58mass% Bi alloy, which has a low melting temperature of 412 K, presents several advantages over other types of solders in low-temperature soldering applications, because it can protect electronic devices from thermal damage under high reflow temperatures. The Sn–Bi Pb-free solder alloys are characterized by a relatively high tensile strength and good creep resistance [11,12,13]. However, because Sn–Bi-based solders also show frangibility and poor ductility, their applications in the packaging industry are limited [14,15].

Recently, several research groups have focused on manufacturing new alloys doped with micro-/nanometer-sized particles, e.g., Ni, Fe, Zn, Ag, Al, ZrO_2_, Al_2_O_3_, TiO_2_, etc., to enhance the mechanical properties and wettability of Pb-free solders for green electronic devices [16,17,18,19,20,21,22]. Takao et al. [23], for instance, reported that Sn–Bi and Sn–Bi–Cu alloys exhibit superplasticity. However, the superplasticity mechanism of Sn-based alloys has not been clearly established. In previous studies, the tensile behavior of Sn–Bi–Cu and Sn–Bi–Ni alloys has been investigated, and the corresponding results indicated that these alloys show superplasticity at high temperatures and low strain rates [24,25]. Cu and Ni occupy a small solid-solution region in the Sn phase diagram and easily form an intermetallic compound with the metal. However, the effect of elements occupying large solid-solution regions in the phase diagrams of Sn or Bi on the superplastic deformation of the resultant alloy has not yet been elucidated. The present study was conducted to investigate the effect of Sb and Zn addition on the microstructures and tensile behavior of Sn-40mass% Bi alloys. Tensile tests were then performed under various temperatures and strain rates to examine the superplastic behavior of the modified alloys. To confirm the superplastic deformation, the concentration of the added element and evolution of microstructures were also evaluated.

## 2. Materials and Methods

**Figure 1:**
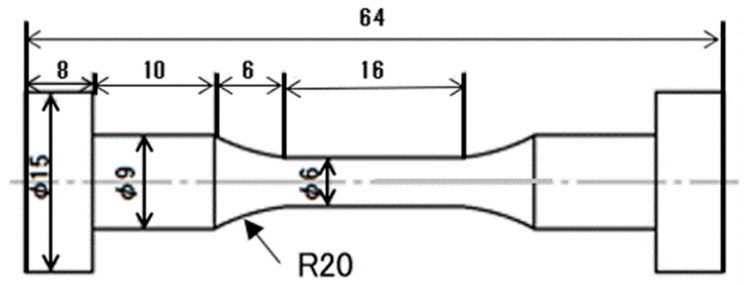
Schematic of the tensile test specimen (unit: mm), adapted from [25].

Sn, Sn-57mass% Bi, Sn-5mass% Sb, and Sn-9mass% Zn (purity, 99.5 mass%) ingot bars were used to synthesize Sn-40mass% Bi-X mass% Sb (X = 0.1, 0.5, and 1.0) and Sn-40mass% Bi-Y mass% Zn (Y = 0.1, 1.0, and 3.0) (the compositional unit “mass%” is omitted hereafter for convenience). Appropriate amounts of the initial ingots were weighed, placed in an Al_2_O_3_ crucible, fused in an electric furnace at 673 K, and then left to solidify for 24 h to achieve a homogeneous composition. The fused ingots were then melted at 653 K, cast in an Al mold, and cooled at a rate of approximately 15 K·min^−1^ to form a cylindrical ingot. Finally, dog-bone-type specimens (Figure 1) were machined from the cylindrical Sn-40Bi-based alloy ingots for the tensile tests.

The tensile tests were performed on a universal material testing machine at strain rates of 5.25 × 10^−2^, 5.25 × 10^−3^, and 5.25 × 10^−4^ s^−1^. Each test piece was exposed to various temperatures of 298, 313, 333, and 353 K in a controlled-atmosphere furnace until complete fracture occurred. In this paper, measurements obtained over three trials under each set of conditions were statistically averaged and reported as the tensile test results.

Specimens were embedded in resin and cut for microstructural observation before and after the tensile testing. The surfaces of the specimens were polished first with SiC papers of up to 1500 grit and then with a 1 μm diamond abrasive and colloidal silica to achieve a mirror-like finish. The cross-sectional and vertical-sectional microstructures and fracture surfaces of the specimens were observed using an optical microscope and a scanning electron microscope (SEM). The crystal orientations were determined by electron backscatter diffraction analysis, and the elemental distributions were evaluated by electron probe microanalysis (EPMA). The solidus and liquidus temperatures of the solder alloys were measured using differential scanning calorimetry (DSC).

## 3. Results and Discussion

### 3.1. Microstructures

**Figure 2:**
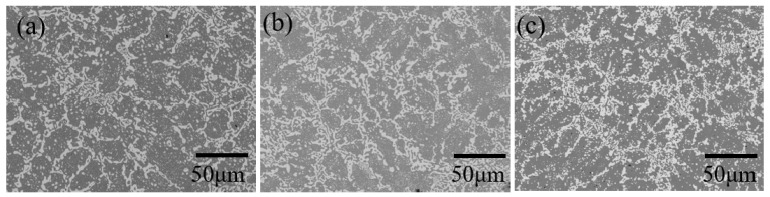
BSE images of (**a**) Sn-40Bi-0.1Sb, (**b**) Sn-40Bi-0.5Sb, and (**c**) Sn-40Bi-1.0Sb.

**Figure 3:**
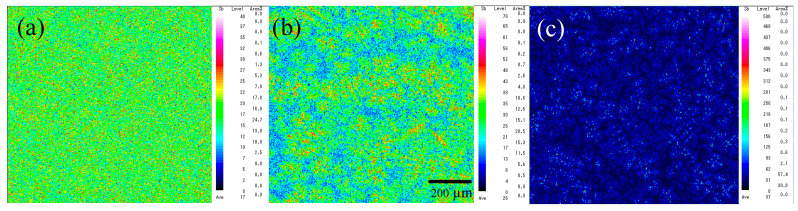
EPMA mapping images of Sb in (**a**) Sn-40Bi-0.1Sb, (**b**) Sn-40Bi-0.5Sb, and (**c**) Sn-40Bi-1.0Sb.

Figure 2 shows the backscattered electron (BSE) images of the Sn–Bi alloys containing various amounts of Sb. In the images, the dark regions represent the solidified β-Sn matrix, and the bright regions reflect the Bi phase dispersed in the β-Sn matrix. The Sn-40Bi-Sb alloys show a hypoeutectic structure composed of primary Sn dendrites with an average diameter of ~20 μm and eutectic Sn–Bi phases with an average diameter of ~5 μm. The addition of Sb to the Sn-40Bi alloys only slightly affected the grain size of the primary Sn phase and the overall alloy microstructure. EPMA of the alloys (Figure 3) revealed that the Sb atoms preferentially exist in the primary Sn phase.

The results of the quantitative EPMA are summarized in Table 1. Sn-40Bi-1.0Sb showed a relatively high Sb concentration (2.4 mass%), as well as a preferential segregation of the Sb atoms in the primary Sn phase (Figure 3c). Further, an increase in the Sb concentration also led to the dissolution of Sb atoms in the eutectic Sn–Bi matrix.

**Figure 4:**
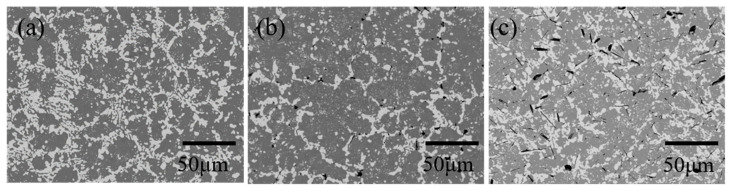
BSE images of (**a**) Sn-40Bi-0.1Zn, (**b**) Sn-40Bi-1.0Zn, and (**c**) Sn-40Bi-3.0Zn.

**Figure 5:**
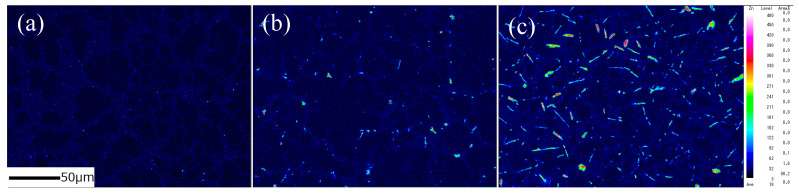
EPMA mapping images of Zn in (**a**) Sn-40Bi-0.1Zn, (**b**) Sn-40Bi-1.0Zn, and (**c**) Sn-40Bi-3.0Zn.

Figure 4 shows the microstructures of the Sn-40Bi-Zn alloys. The EPMA results presented in Figure 5 reveal that the alloys consist of a gray β-Sn phase, a eutectic Sn–Bi phase, and a needle-like Zn-rich phase. A significant increase in the Zn-rich phases with increasing Zn concentration was observed. The EPMA further confirmed that the Zn atoms preferentially existed in the Bi phase of the eutectic Sn–Bi matrix. This result agrees with those of Mokhtari et al. [26] and Hirata et al. [27]. The grain size of the primary Sn phase of Sn-40Bi-1.0Zn, shown in Figure 4b, was larger than that of the primary Sn phase of the other Sn–Bi–Zn alloys. The EPMA of the Zn-rich phase showed that the phases were mainly composed of Zn, and the Zn mass percentage increased to more than 50%, as shown in Figure 5b,c.

### 3.2. Tensile Properties

**Figure 6:**
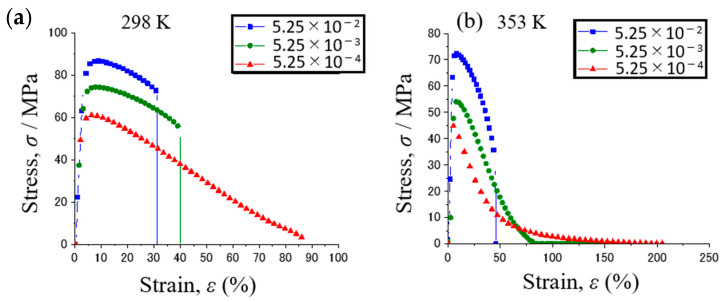
Stress–strain curves of Sn-40Bi-0.1Sb under various strain rates at (**a**) 298 and (**b**) 353 K.

Figure 6 shows the stress–strain curves of Sn-40Bi-0.1Sb at various strain rates and temperatures (298 and 353 K). At 298 K, the alloy exhibited a nearly steady-state flow as the stress approached its yield strength. The tensile strength of the specimen decreased, whereas its elongation increased with the decreasing strain rate. The tensile behavior of Sn-40Bi-0.1Sb did not reflect superplasticity at 298 K. At 353 K, the alloy exhibited a gradual decrease in tensile strength under strain rates of 5.25 × 10^−2^ and 5.25 × 10^−3^ s^−1^. The specimen fractured when the stress reached its yield strength. Under a strain rate of 5.25 × 10^−4^ s^−1^, the yield strength of the alloy exhibited an initial sharp decrease, followed by a more gradual decrease until rupture occurred. The greatest elongation of this specimen observed under the conditions of 353 K and 5.25 × 10^−4^ s^−1^ was 206%. Superplastic behavior is defined as the ability to withstand a strain of >200% [28,29]. Sn-40Bi-0.1Sb demonstrated superplasticity at a temperature of 353 K and a strain rate of 5.25 × 10^−4^ s^−1^.

**Figure 7:**
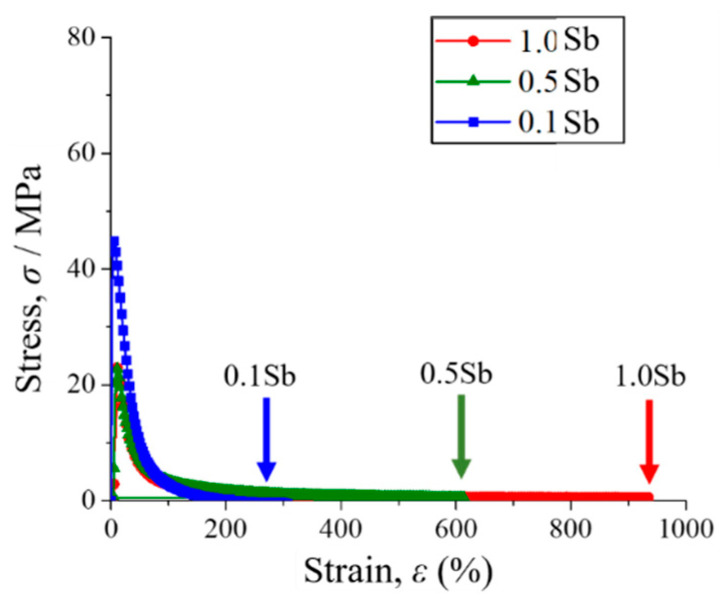
Stress–strain curves of various Sn-40Bi-Sb alloys at 353 K under a strain rate of 5.25 × 10^−^^4^ s^−^^1^.

**Figure 8:**
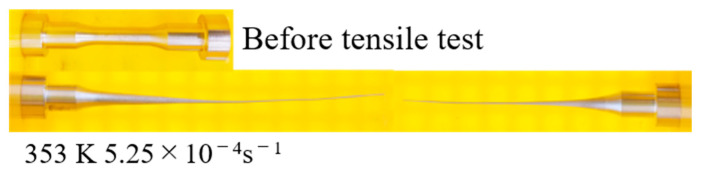
Photographs of Sn-40Bi-1.0Sb before and after the tensile testing at 353 K under a strain rate of 5.25 × 10^−^^4^ s^−^^1^.

**Figure 9:**
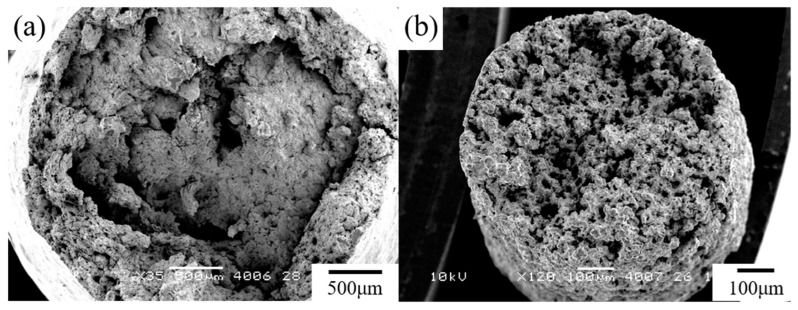
Fractured surface of the Sn-40Bi-1.0Sb alloy after the tensile test under a strain rate of 5.25 × 10^−4^ s^−1^ at (**a**) 298 K and (**b**) 353 K.

In general, the stress–strain curves of the Sn-40Bi-Sb alloys are similar to those of Sn-40Bi-0.1Cu and Sn-40Bi-0.01Ni [24,25]. Figure 7 shows the stress–strain curves of the Sn-40Bi-Sb alloys at 353 K under a strain rate of 5.25 × 10^−4^ s^−1^. The highest tensile strength (45 MPa) under these conditions is observed in Sn-40Bi-0.1Sb. The tensile strength of the alloys decreases with increasing Sb concentration. Indeed, the tensile strength of Sn-40Bi-1.0Sb, at 23 MPa, is only half of that of Sn-40Bi-0.1Sb at 353 K under a strain rate of 5.25 × 10^−4^ s^−1^. Conversely, the elongation increases with the increasing Sb concentration. The maximum elongation of Sn-40Bi-1.0Sb under the conditions of 353 K and 5.25 × 10^−4^ s^−1^ is no less than 900%, as illustrated in Figure 8. The specimens generally exhibited longer elongation, without necking, and chisel-point fractures as their fracture mode. This result is obtained possibly because Sb is solid-solution not only in the primary Sn phase but also in the Bi phase. Therefore, in contrast to Cu and Ni, which are typically added in low amounts, a larger amount of Sb must be added to the alloy to achieve superplastic deformation with long elongation. The microstructural observations further indicated that the superplastic deformation is likely to occur in the Sn-40Bi-Sb alloys, because the structures of the primary Sn phase and eutectic Sn–Bi matrix become finer as the Sb concentration increases. Figure 9 shows the fractograph of Sn-40Bi-1.0Sb after the tensile test under a strain rate of 5.25 × 10^−4^ s^−1^ at 298 and 353 K. At 298 K, the alloy displays a ductile fracture mode, whereas, at 353 K, it displays a chisel-point fracture mode. The area of the Sn-40Bi-1.0Sb alloy at 353 K was reduced by approximately 98%. Thus, the reduction in the area and elongation of the Sn-40Bi-Sb alloys increases with an increasing concentration of Sb.

**Figure 10:**
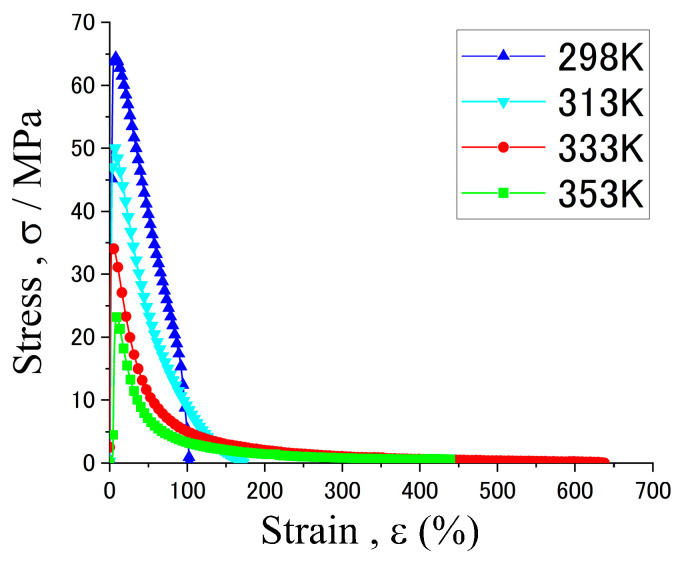
Stress–strain curves of Sn-40Bi-0.1Zn under a strain rate of 5.25 × 10^−^^4^ s^−^^1^ at various temperatures.

Figure 10 shows the stress–strain curves of Sn-40Bi-0.1Zn at various temperatures under a strain rate of 5.25 × 10^−4^ s^−1^. The ductility of Sn-40Bi-0.1Zn exhibited gradual improvements as the temperature increased. The specimen showed superplastic deformation at temperatures above 333 K and elongation of up to 630%. Furthermore, specimens with different Zn concentrations demonstrated large elongations at 333 and 353 K. The Sn-40Bi-Zn alloys show the same temperature and strain-rate dependences as the Sn-40Bi-Sb alloys (Figure 6); thus, the stress–strain curves in various conditions are omitted for this alloy in this paper.

**Figure 11:**
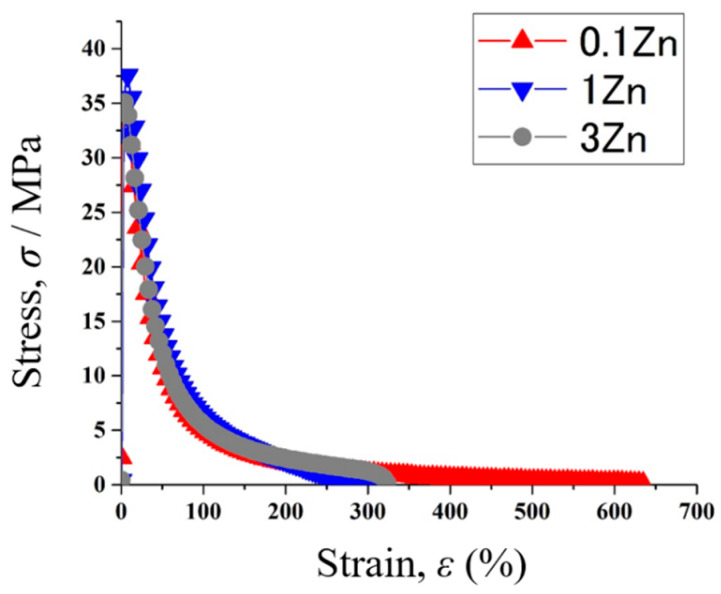
Stress–strain curves of various Sn-40Bi-Zn alloys at 333 K under a strain rate of 5.25 × 10^−^^4^ s^−^^1^.

Figure 11 shows the stress–strain curves of the various Sn-40Bi-Zn specimens at 333 K, under a strain rate of 5.25 × 10^−4^ s^−1^. The elongation of the alloys increased in the order of 1Zn < 3Zn < 0.1Zn, likely because their microstructures were modified by Zn addition (Figure 4). Interestingly, the Sn-40Bi-Zn alloys showed extensive crystallization of the needle-like Zn particles when the metal was added at a rate of 1 mass% or higher. Therefore, the elongations of Sn-40Bi-1Zn and Sn-40Bi-3Zn were lower than that of Sn-40Bi-Zn. The fractured surfaces of the Sn-40Bi-Zn alloys after the tensile test are almost the same as those of the Sn-40Bi-Sb alloys (Figure 9). Thus, there is no difference between the additional elements on the fractured photographs.

**Figure 12:**
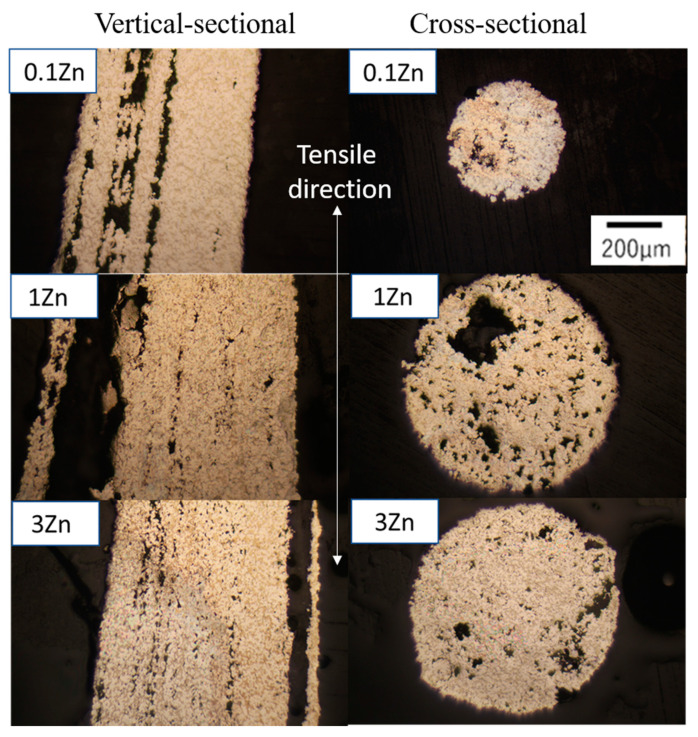
Vertical and cross-sectional optical microscopic images of various Sn-40Bi-Zn alloys after the tensile tests.

Figure 12 shows the vertical and cross-sectional images of various Sn-40Bi-Zn alloys after the tensile tests. In these tests, the tensile stress was applied to the specimens in the vertical direction. All the alloys displayed chisel-point fractures, which indicate superplastic deformation. The area of the Sn-40Bi-0.1Zn alloy at 353 K was reduced by approximately 99.5%, indicating that the reduction in the area and elongation of the Sn-40Bi-Zn alloys at 353 K decreased with the increasing Zn concentration. The number of voids in the specimens clearly increased with the increasing Zn concentration. Whether there is a correlation between the needle-like Zn particles and the number of voids is unknown, but the tendency of the elongation to decrease with the increasing Zn concentration is considered to be related to the number of voids in the specimens. At high temperatures and low strain rates, which are conducive to superplastic behavior, specimens do not show remarkable elongation of the primary Sn phase in the tensile direction; cracks and voids are observed to be segregated at the grain boundaries around the intermetallic compounds [25]. These results indicate that void accumulation begins from the intermetallic compounds or metal particles, and the final structure of the alloy may be different from its initial dendritic structure. Therefore, dynamic recovery as well as dynamic recrystallization occur after the ultimate tensile strength is achieved, and grain boundary slip deformation may be accompanied by diffusion creep due to void accumulation. Thus, the deformation of the primary Sn phase is the dominant deformation mechanism of the Sn-40Bi-Zn alloys at low temperatures and high strain rates. At high temperatures and low strain rates, the deformation of these alloys leads to dynamic recovery, dynamic recrystallization, and/or grain boundary slip.

Slip deformation may be speculated to be the dominant deformation mechanism of the eutectic Sn–Bi–Zn matrix. Table 2 summarizes the solidus and liquidus temperatures of the Sn-40Bi and Sn-40Bi-Zn alloys obtained from DSC measurements. The melting point temperature of the alloys decreased when the amount of Zn added was equal to or exceeded 1 mass%, and it was 406 K when the added Zn amount was 3 mass%. The liquidus temperature increased from 440 K in the alloy without Zn to 452 K in the alloy with 1 mass% Zn, but it decreased sharply to 427 K in the alloy with 3 mass% Zn, likely because the ternary eutectic composition of the alloy is close to that of Sn-40Bi-3Zn. Because the tensile test temperature (353 K) is only 87% of the solidus temperature of Sn-40Bi-3Zn, the elongation of this alloy may be estimated to be larger than that of Sn-40Bi-1Zn. This is because superplastic deformation occurring at high temperatures and low liquidus temperatures tends to promote plastic flow.

### 3.3. Strain Rate Sensitivity Index (m)

**Figure 13:**
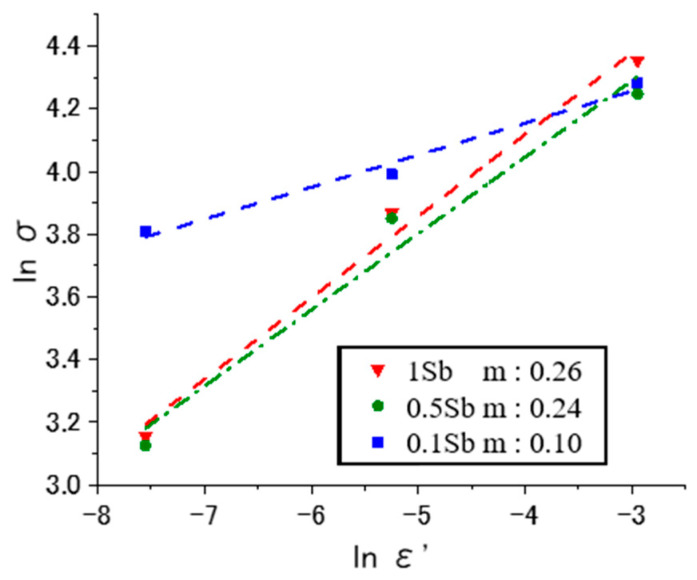
Strain rate sensitivity index (*m*) of the Sn-40Bi-Sb alloys at 353 K (σ, stress; $\dot{\varepsilon }$, strain rate).

Figure 13 shows the strain rate sensitivity index (*m*) of various Sn-40Bi-Sb alloys at 353 K. In this study, *m* was generally defined by Equation (1):

(1) $$m=\frac{d\ln\sigma }{d\ln\dot{\varepsilon }},$$

where σ is the stress, and $\dot{\varepsilon }$ is the strain rate. The *m* value is an important parameter that describes the high-temperature tensile ductility of an alloy material; it is calculated from the slopes of the lines obtained at various temperatures and increases with increasing temperature. For materials without superplasticity, *m* < 0.2, whereas, for general superplastic materials with fine grains, the *m* value is usually very large, i.e., *m* = 0.3–1.0. A large *m* value indicates that large superplastic elongation can be obtained [30,31,32]. The *m* value calculated from the slopes of the lines shown in Figure 13 is lower than the critical value of 0.3, indicating that the alloys are characterized by fine-grained superplasticity. This result is similar to previous findings reported for Sn-40Bi-0.1Cu and Sn-40Bi-0.01Ni [24,25]. Because the microstructures and average grain sizes of the Sn-40Bi-Sb alloys are similar to those of Sn-40Bi-0.1Cu, the *m* value of the Sn-40Bi-Sb alloys also increases with the increasing Sb concentration. Evidently, increasing the Sb concentration influences the superplastic-like deformation of the resultant alloys. These results suggest that although the Sn-40Bi-Sb alloys show superplastic-like deformation, their deformation mechanism is not based on fine-grained superplasticity. Thus, it can be considered that the deformation mechanism of the Sn-40Bi-X alloy might be caused by the grain boundary slip, diffusion creep, and dislocation creep.

## 4. Conclusions

In this study, the microstructures and tensile behaviors of the Sn-40Bi-X% Sb (X = 0.1, 0.5, 1.0) and Sn-40Bi-Y% Zn (Y = 0.1, 1.0, 3.0) alloys at various temperatures and strain rates were investigated using SEM and tensile tests. The following results were obtained.

(1) The tensile strength decreased and the elongation increased with the increasing temperature or decreasing strain rate.
(2) The Sn-40Bi-Sb and Sn-40Bi-Zn alloys demonstrated superplasticity at high temperatures (>333 K) and low strain rates (<5.25 × 10^−3^ s^−1^).
(3) The *m* value of the alloys increased with the increasing temperature. Moreover, the *m* value of the Sn-based alloys was lower than the critical value of 0.3, implying that these alloys did not exhibit fine-grained superplasticity but superplastic-like deformation with grain boundary slip and diffusion creep. The maximum *m* value of Sn-40Bi-1Sb was 0.26.
(4) The Sb atoms were dissolved in both the primary Sn phase and the eutectic Sn–Bi matrix. In contrast, the Zn atoms were dissolved in the eutectic Sn–Bi matrix, and needle-like Zn crystals were formed when the Zn concentration exceeded 1 mass%. The deformation of the primary Sn phases is the dominant deformation mechanism at low temperatures and high strain rates. Moreover, deformation at high temperatures and low strain rates leads to recovery, dynamic recrystallization, and/or grain boundary slip.

Further research on complex Sn–Bi-based solder alloys will be conducted in the future to obtain more low-temperature solder candidates that could be applied to electronic devices.

## Figures and Tables

**Table 1:** EPMA results of the Sn-40Bi-Sb alloys.

Specimen	Sb Concentration (Mass %)	
	Primary Sn Phase	Eutectic Sn–Bi Matrix
Sn-40Bi-0.1Sb	0.09	0
Sn-40Bi-0.5Sb	1.0	0.16
Sn-40Bi-1.0Sb	2.4	0.41

**Table 2:** Solidus and liquidus temperatures of the Sn-40Bi-Zn alloys.

Specimen	Solidus Temperature (K)	Liquidus Temperature (K)
Sn-40Bi	411	440
Sn-40Bi-0.1Zn	411	445
Sn-40Bi-1Zn	406	452
Sn-40Bi-3Zn	406	427

## Data Availability

All data are available from the corresponding author upon reasonable request.

## References

[1] Suganuma K. (2001). Advances in lead-free electronics soldering. Curr. Opin. Solid State Mater. Sci..

[2] Synedlina J.J., Nurmib S.T., Lepisto T.K., Ristolainen E.O. (2006). Mechanical and microstructural properties of SnAgCu solder joints. Mater. Sci. Eng. A.

[3] Gain A.K., Zhang L. (2016). Growth mechanism of intermetallic compound and mechanical properties of nickel (Ni) nanoparticle doped low melting temperature tin-bismuth (Sn-Bi) solder. J. Mater. Sci. Mater. Electron..

[4] Wu M.L., Yu D.Q., Law C.M.T., Wang L. (2002). The properties of Sn-9Zn lead-free solder alloys doped with trace rare earth elements. J. Electron. Mater..

[5] Gain A.K., Zhang L., Quadir M.Z. (2016). Thermal aging effects on microstructures and mechanical properties of an environmentally friendly eutectic tin-copper solder alloy. Mater. Des..

[6] Sahin M., Cadirli E. (2012). The effects of temperature gradient and growth rate on the microstructure of directionally solidified Sn-3.5Ag eutectic solder. J. Mater. Sci. Mater. Electron..

[7] Matijasevic S., Lee C.C., Wang C.Y. (1993). Au-Sn alloy phase diagram and properties related to its use as a bonding medium. Thin Solid Films.

[8] Gain A.K., Zhang L. (2016). Interfacial microstructure, wettability and material properties of nickel (Ni) nanoparticle doped tin-bismuth-silver (Sn-Bi-Ag) solder on copper (Cu) substrate. J. Mater. Sci. Mater. Electron..

[9] Hsi S., Lin C.T., Chang T.C., Wang M.C., Liang M.K. (2010). Interfacial Reactions, Microstructure, and Strength of Sn-8Zn-3Bi and Sn-9Zn-Al Solder on Cu and Au/Ni (P) Pads. Metall. Mater. Trans. A.

[10] Gain A.K., Zhang L. (2016). Harsh service environment effects on the microstructure and mechanical properties of Sn–Ag–Cu-1 wt% nano-Al solder alloy. J. Mater. Sci. Mater. Electron..

[11] Tomlinson W.J., Collier I. (1987). The mechanical properties and microstructures of copper and brass joints soldered with eutectic tin-bismuth solder. J. Mater. Sci..

[12] Mei Z., Morris J.W. (1992). Characterization of eutectic Sn-Bi solder joints. J. Electron. Mater..

[13] Yebisuya T., Kawakubo T. (1993). Creep and tensile properties of cast Bi-Sn, Bi-Pb and Bi-Sn-Pb solders. J. Jpn. Inst. Met. Mater..

[14] Watanabe H. (2005). The lead-free solder of addition micro-elements in industrial products. J. Jpn. Inst. Electron. Packag..

[15] Nagano M., Hidaka N., Watanabe H., Shimoda M., Ono M. (2006). Effect of additional elements on creep properties of the Sn-Ag-Cu lead free solder. J. Jpn. Inst. Electron. Packag..

[16] Mccormack M., Chen H.S., Kammlott G.W., Jin S. (1997). Significantly improved mechanical properties of Bi-Sn solder alloys by Ag-doping. J. Electron. Mater..

[17] Sakuyama S., Akamatsu T., Uenishi K., Sato T. (2009). Effects of a third element on microstructure and mechanical properties of eutectic Sn-Bi solder. Trans. Jpn. Inst. Electron. Packag..

[18] Okamoto K., Nomura K., Doi S., Akamatsu T., Sakuyama S., Uenishi K. (2013). Effect of Sb and Zn addition on impact resistance improvement of Sn-Bi solder joints. Int. Symp. Microelectron..

[19] Zhang L., Tu K.N. (2014). Structure and properties of lead-free solders bearing micro and nano particles. Mater. Sci. Eng. R.

[20] Gain A.K., Zhang L. (2019). Effects of Ni nanoparticles addition on the microstructure, electrical and mechanical properties of Sn-Ag-Cu alloy. Materialia.

[21] Tsao L.C., Chang S.Y. (2010). Effects of Nano-TiO_2_ additions on thermal analysis, microstructure and tensile properties of Sn3.5Ag0.25Cu solder. Mater. Des..

[22] Hirata A., Shoji I., Tsuchida T., Ookubo T. Effect of electrode material on joint strength of soldered joints with Sn-Bi and Sn-Bi-Sb lead-free solder balls. Proceedings of the ASME 2013 International Technical Conference and Exhibition on Packaging and Integration of Electronic and Photonic Microsystems.

[23] Takao H., Yamada A., Hasegawa H., Matsui M. (2002). Mechanical properties and solder joint reliability of low-melting Sn-Bi-Cu lead free solder alloy. J. Jpn. Inst. Electron. Packag..

[24] Yamauchi A., Ida K., Fukuda M., Yamaguchi T. (2018). Tensile properties of Sn-Bi lead-free solder alloys. Solid State Phenom..

[25] Umeyama J., Yamauchi A. (2019). Tensile behavior and superplastic deformation of Sn-Bi-Cu Alloy. Mater. Trans..

[26] Mokhtari O., Zhou S., Chan Y.C., Nishikawa H. (2016). Effect of Zn addition on interfacial reactions between Sn-Bi solder and Cu substrate. Mater. Trans..

[27] Hirata Y., Yang C., Lin S., Nishikawa H. (2021). Improvements in mechanical properties of Sn-Bi alloys with addition of Zn and In. Mater. Sci. Eng. A.

[28] Ohsawa H., Nishimura H. (1989). Manufacturing method of superplastic materials and commercial applications. J. Jpn. Inst. Light Met..

[29] (2002). Glossary of Terms Used in Metallic Superplastic Materials.

[30] Maruyama K., Nakashima H. (1997). Materials Science for High Temperature Strength.

[31] Nieh T.G., Wadsworth J., Sherby O.D. (1997). Superplasticity in Metals and Ceramics.

[32] Ridley N., Giuliano G. (2011). Metals for superplastic forming. Superplastic Forming of Advanced Metallic Materials: Methods and Applications.

